# Stereotactic Body Radiotherapy for Metastatic Lung Cancer as Oligo-Recurrence: An Analysis of 42 Cases

**DOI:** 10.1155/2012/454107

**Published:** 2012-10-04

**Authors:** Wataru Takahashi, Hideomi Yamashita, Yuzuru Niibe, Kenshiro Shiraishi, Kazushige Hayakawa, Keiichi Nakagawa

**Affiliations:** ^1^Department of Radiology, University of Tokyo Hospital, 7-3-1, Hongo, Bunkyo-ku, Tokyo 113-8655, Japan; ^2^Department of Radiology and Radiation Oncology, Kitasato Universtiy, Kanagawa 252-0374, Japan

## Abstract

*Purpose*. To investigate the outcome and toxicity of stereotactic body radiotherapy (SBRT) in patients with oligo-recurrence cancer in the lung (ORCL). *Methods and Materials*. A retrospective review of 42 patients with ORCL who underwent SBRT in our two hospitals was conducted. We evaluated the outcome and adverse effects after SBRT for ORCL. *Results*. All patients finished their SBRT course without interruptions of toxicity reasons. The median follow-up period was 20 months (range, 1–90 months). The 2-year local control rate and overall survival were 87% (95% CI, 75–99%) and 65% (95% CI, 48–82%). As for prognostic factor, the OS of patients with a short disease-free interval (DFI) <31.9 months, between the initial therapy and SBRT for ORCL, was significantly worse than the OS of long DFI ≧31.9 months (*P* < 0.05). The most commonly observed late effect was radiation pneumonitis. One patient had grade 4 gastrointestinal toxicity (perforation of gastric tube). No other ≧ grade 3 acute and late adverse events occurred. There were no treatment-related deaths during this study. *Conclusions*. In patients with ORCL, radical treatment with SBRT is safe and provides a chance for long-term survival by offering favorable local control.

## 1. Introduction

Lung is one of the common sites of metastasis after definitive therapy for a primary cancer. So far, recurrent or metastatic lung cancers have been considered to uniformly carry a poor prognosis because multiple metastases tend tobe difficult to treat intensively.Chemotherapy has been broadly applied as a standard managementat these conditions. On the other hand, the innovation of methods of early detection of recurrence, such as positron-emission tomography (PET), allows the detection of limited site recurrent, called oligo-recurrence. Oligo-recurrence, proposed by Niibeet al.in 2006 [1–4], was the condition of one or a few metastatic or recurrent lesions occurred with controlled primary lesion. For case with oligo-recurrence cancer in the lung (ORCL), the controversy exists regarding the optimal approach of these metastatic sites. Despite surgical approach is considered as an alternative for a single metastasis, there are many patients with ORCL who were not amenable for metastasectomy. For them, less invasive techniques such as SBRT have been used to treat ORCL. In cases considered to have a favorable prognosis, radical treatment with SBRT seems to be beneficial for prolonging the survival time. However, the role of radiotherapy and the prognostic factors for oligo-recurrencehave not yet been clearly elucidated [5]. In this study, we evaluated the efficacy and toxicity of SBRT for patients with oligo-recurrence cancer treated from 2001 through 2011 in two hospitals.

## 2. Materials and Methods

### 2.1. Patient Eligibility and Pretreatment Evaluation

A retrospective review of all patients with ORCL treated with SBRT after prior therapy at University of Tokyo Hospital and Kitasato University Hospital from April 2001 to July 2011 was conducted. Patients with ORCL who were not suitable for surgery due to medical or functional reasons were included in this analysis. Pretreatment evaluation included a complete medical history, physical examination, computed tomography (CT), pulmonary function tests, and laboratory tests. In addition, 36 of 42 patients (86%) were evaluated with ^18^F fluorodeoxyglucose (FDG)-PET before treatment. Inclusion criteria of this study were as follows: (a) primary cancer was completely treated; (b) the number of lung metastases were up to three; (c) there was no other distant metastasis or other distant metastasis was scheduled to be treated with curative intent after SBRT. As long as these evaluations fulfilled the inclusion criteria, there was no restriction regarding tumor size, location, or general pulmonary function. Radiotherapy was the exclusive treatment modality in all patients.

### 2.2. Radiotherapy

SBRT was given with 6 MV X-ray of a linear accelerator. In curative intention, hypofractionated SBRT was delivered to a median dose of 48 Gy (range, 20–56 Gy) with a median daily dose of 12 Gy (range, 8–30 Gy). Dose and fractionation schedules were chosen depending on location and institution. In University of Tokyo Hospital, SBRT was performed using the Synergy linear accelerator (ELEKTA), which fully integrates IGRT by means of kV-CT scanning. In Kitasato University Hospital, real-time tumor-tracking radiotherapy was used for SBRT. The gross tumor volume (GTV) or internal target volume (ITV) included the visible gross tumor mass on CT were delineated on a three-dimensional radiation treatment planning system (3D RTPS) using the lung window. The planning target volume (PTV) was created by adding five mm margin to the ITVs in all directions.

### 2.3. Follow-Up

After completion of therapy, patients were scheduled for regular follow-up visits 3 monthly during the first year, 6 monthly thereafter. Those who did not appear for a routine follow-up were contacted by phone. Follow-up evaluations included a history and physical examination and CT scans of the thorax. Additional imaging investigations such as FDG-PET were only required if there was clinical suspicion of recurrence. In this study, we define local recurrence as an increase in opacity size on CT imaging, along with either increased maximum standardized uptake values (SUVmax) ≧5 on FDG-PET, or biopsy proof of disease [6]. Toxicity was evaluated and scored according to the National Cancer Institute Common Terminology Criteria for Adverse Events (NCI CTCAE) version 4.0, with toxicity occurring within 3 months after the initiation of RT classified as acute toxicity. Late toxicity was graded using the RTOG/EORTC criteria.

### 2.4. Statistical Analysis

Thebaseline follow-up date was the first day of radiotherapy, and the last follow-up date was the last Hospital visit or phone day. Overall survival (OS) was calculated from the start of the SBRT to the date of death, censoring the last follow-up date. Local control rate (LCR) was calculated from the start of the SBRT to the first local recurrencedate, censoring death or last follow-up date. 

To discuss risk factors for OS and LCR, the patients of ORCL were classified into two groups: early recurrence group and late recurrence group. The former group consisted of 21 patients whose disease-free interval (DFI), meaning interval between the start date of initial therapy and the start date of SBRT for ORCL, was shorter than 31.9 months (median DFI time). In addition, we compared OS and LCR following SBRT for ORCL from colorectal cancer (CRC) and other origins. OS and LCR curves were plotted using the Kaplan-Meier method. Log-rank testing was used to compare OS and LCR between the subsets of patients analyzed. All analyses were performed using SPSS software version 12.0 (SPSS Inc., Chicago, IL).

## 3. Results

From April 2001 to July 2011, we identified 42 patients with ORCL who were treated with SBRT. The median age was 69 years (range, 25–84 years). There were 30 men and 12 women. The median maximum diameter of metastatic tumor was 19 mm (range, 9–40 mm). Patient characteristics are shown in Table 1. One patient underwent chemotherapy for ORCL before SBRT and the other 41 patients did not undergo neoadjuvant, concurrent, or adjuvant chemotherapy for ORCL. Sites of primary disease included lung (*n* = 16), colon and rectum (*n* = 7), head and neck (6), esophagus (*n* = 4), uterus (*n* = 4), kidney (*n* = 2), and others (renal pelvis, breast, sarcoma; *n* = 3). Of these, 32 patients had lung metastasis alone, 8 patients had another lung metastasis treated with SBRT after initial SBRT, and 2 patients had a distant metastasis in addition to lung lesion (retroperitoneal node and adrenal gland). These distant metastases in both patients were also treated with SBRT after completing SBRT for lung lesion. At the time to analysis, they were alive without evidence of any recurrence.

All patients finished their SBRT course without interruptions of toxicity reasons. Acute toxicities were mild and tolerable except for one case. Grade 4 acute adverse event were observed in only 1 patient (2%), which displayed the perforation of the pulled-up gastric tube. This patient was a 59-year-old man, with esophageal cancer after total esophagectomy with esohageal replacement by means of a gastric tube, had undergone SBRT, consisting of 50 Gy in four fractions in 4 days. The D2 cc, the minimum dose in the most irradiated 2 cc of the gastric tube, was 48.66 Gy. He was a heavy smoker and had an alcohol problem. Two months later, he developed perforation of the gastric tube.

**Figure 1:**
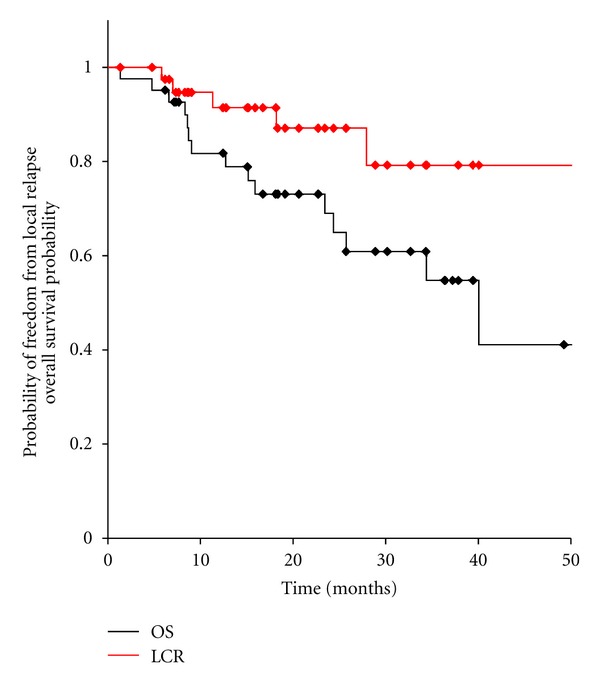
Overall survival and local control of 42 patients with oligo-recurrence cancer in the lung.

No other grade ≥3 acute side effects occurred. Twenty-one patients (50%) and 5 patients (12%) experienced grade 1 and 2 adverse event after irradiation of metastases, respectively. Of the 42 patients, 21 patients (50%) and 3 patients (7%) displayed grade 1 pneumonitis (asymptomatic, radiographic findings only) and grade 2 pneumonitis (symptomatic, not interfering with activities of daily living), respectively. No grade ≥3 late adverse events occurred until now. The medianduration of follow-up was 20 months (range, 1–90 months) for all patients and 24 months (range, 6–90 months) for those alive. The 1- and 2-year local control rates were 91% (95% CI, 82–100%) and 87% (95% CI, 75–99%), respectively (Figure 1). At the time of last follow-up, 16 patients had died. The causes of death were recurrence (*n* = 9), other diseases (*n* = 7). The overall 1- and 2-year survival rates were 81% (95% CI, 69–94%) and 65% (95% CI, 48–82%), respectively (Figure 1), with a median survival time of 40 months. Seventeen of 42 patients showed a long-term survival of longer than 2 years.

**Figure 2:**
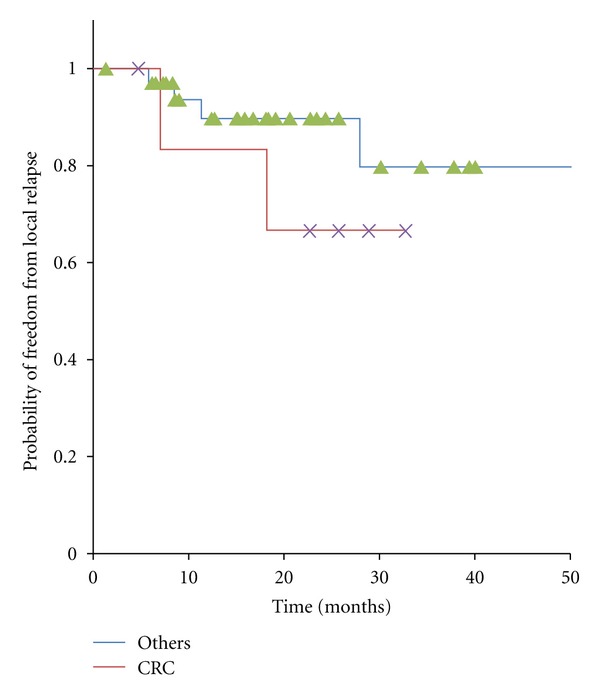
Kaplan-Meier curves for local control in 42 patients with oligo-recurrence cancer in the lung, cancers from colorectal cancer and ones from other origins.

**Figure 3:**
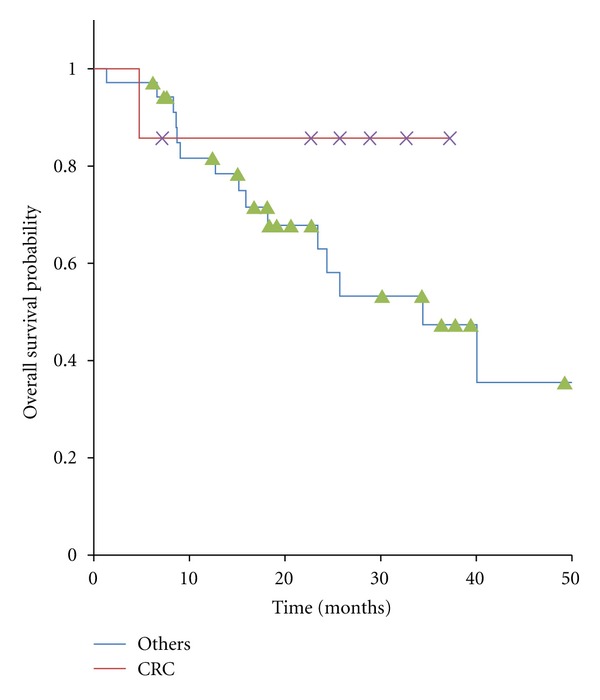
Kaplan-Meier curves for overall survival in 42 patients with oligo-recurrence cancer in the lung, cancers from colorectal cancer and ones from other origins.

In present study, seven patients with ORCL originated from CRC and 35 patients originated from other origins were treated by SBRT. The 1- and 2-year LCR in ORCL from CRC and in ORCL from other origins were 83% and 67%, 89% and 89%, respectively (Figure 2).The overall 1- and 2-year survival rates in ORCL from CRC and in ORCL from other origins were 85% and 85%, 82% and 63%, respectively (Figure 3). These results showed no significant difference in LCR (*P* = 0.31) and OS (*P *= 0.26).

**Figure 4:**
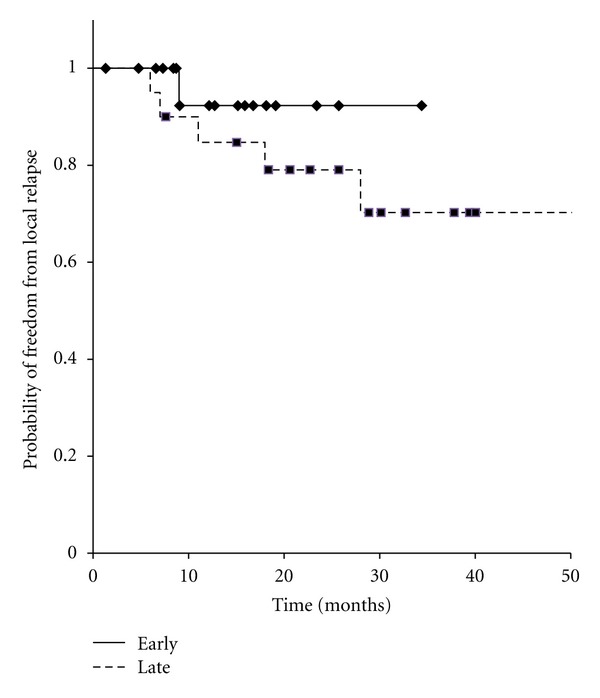
Kaplan-Meier curves for local control in 42 patients with oligo-recurrence cancer in the lung, early recurrence group versus late recurrence group.

**Figure 5:**
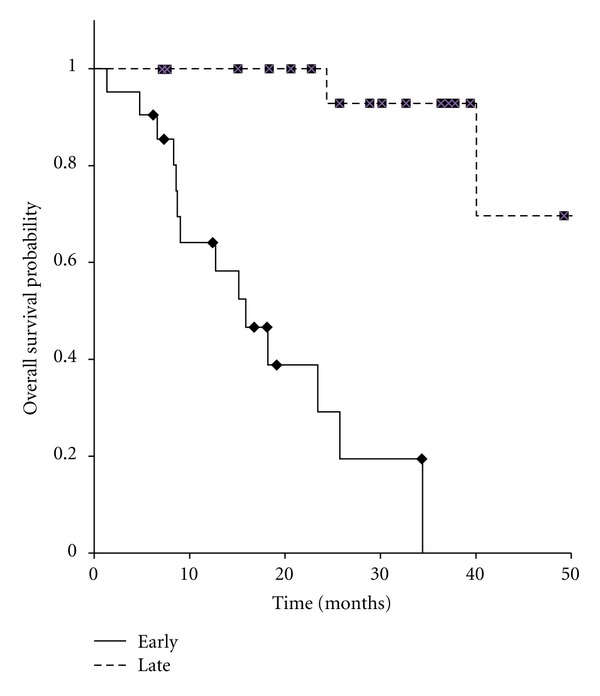
Kaplan-Meier curves for overall survival in 42 patients with oligo-recurrence cancer in the lung, early recurrence group versus late recurrence group.

We also analyzed the LCR and OS differences stratified by DFI divided into <31.9 or ≧31.9 months. As shown in Figure 4, the result indicated a negative correlation between DFI and LCR (*P* = 0.29). On the other hand, early recurrence group (short DFI) had significantly bad prognosis (*P* < 0.05; Figure 5).

## 4. Discussion

Although this is a retrospective study with a limited sample size, our results are also comparable to other studies in ORCL [7–9]. Norihisa et al. [10] also previously showed the results of SBRT for 43 metastatic lung cancers. In their series, the survival rates and local control rate at 2 years were reported to be 84.3% and 90%, respectively. Ricardiet al. [11] also reported a study of SBRT for oligometastatic lung tumors. Sixty-one patients treated with SBRT achieved 89% in local control and 66.5% in survival at 2 years. 

Several studies have now shown that the local control after SBRT for lung metastases from CRC is worse than that from other origins. Takeda et al. [12] reported the difficulty of local control for ORCL from CRC. Norihisaet al. [10] proposed dose escalation in SBRT for CRC patients in order to achieve better local control. In the current study, there was no significant difference between CRC and other origins in LCR (*P *= 0.31) and OS (*P* = 0.26), respectively.

Furthermore, we also analyzed the OS and LCR differences stratified by DFI divided into <31.9 or ≧31.9 months. As shown in Figure 5, short DFI was the prognostic factor (*P* < 0.05). Thus, even as oligo-recurrence, early metastasis may be bad prognostic factor.

It seems from these results that SBRT is an effective and safe treatment for patients with lung metastases as oligo-recurrence. In SBRT for lung metastases, limited toxicity rates are reported by several authors [12]. In our series, there was no patient with serious late toxicities except for one patient with perforation of gastric tube. Although it is likely that the perforation may be caused mainly by radiation to gastric tube, smoking and bad nutrition might have been partly related to this perforation. Several reports advocated that deterioration in smoking and bad nutritional status during radiotherapy could be associated with poorer short-term treatment outcomes and severe side effect [13, 14].

Several limitations of this study warrant mention. First, it was a retrospective review with a limited number of patients and limited follow-up. Second, we treated ORCL from various primary cancers by using different treatment protocol. There was a wide range of doses prescribed, and a variety of fractionation schema.

## 5. Conclusions

In patients with ORCL, radical treatment with SBRT offers good local control and provides a real chance for long-term survival. In addition, even in ORCL, SBRT is a safe and efficacious modality and appears to be well tolerated.

## Figures and Tables

**Table 1:** Patients characteristics (*n* = 42).

Variable	Distribution	No. of patients	%
Sex	Male	30	71
	Female	12	29
Age	Median	69 years	
	Range	25–84 years	
Karnofsky Performance status	Median	90	
	Range	50–90	
Number of metastases	1	32	76
	2	10	24
	≧3	0	0
Maximum diameter (mm)	Median	19 mm	
	Range	9–40 mm	
Primary site	Lung	16	38
	Colon and rectum	7	17
	Head and neck	6	14
	Esophagus	4	10
	Uterus	4	10
	Kidney	2	5
	Other	3	5
Follow-up (months)	Median	20 months	
	Range	1–90 months	
